# Blending Emotions and Cross-Modality in Sonic Seasoning: Towards Greater Applicability in the Design of Multisensory Food Experiences

**DOI:** 10.3390/foods9121876

**Published:** 2020-12-17

**Authors:** Felipe Reinoso-Carvalho, Laura H. Gunn, Enrique ter Horst, Charles Spence

**Affiliations:** 1 School of Management, University of los Andes, Calle 21 1-20, SD building, Room SD-940, Bogotá 111711, Colombia; ea.terhorst@uniandes.edu.co; 2 Department of Public Health Sciences, University of North Carolina at Charlotte, Charlotte, NC 28223, USA; laura.gunn@uncc.edu; 3 School of Data Science, University of North Carolina at Charlotte, Charlotte, NC 28223, USA; 4 Faculty of Medicine, School of Public Health, Imperial College London, London SW7 2AZ, UK; 5 Crossmodal Research Laboratory, University of Oxford, Oxford OX2 6FF, UK; charles.spence@psy.ox.ac.uk

**Keywords:** Bayesian, cross-modal, emotions, multisensory, sonic seasoning, tasting

## Abstract

Sonic seasoning refers to the way in which music can influence multisensory tasting experiences. To date, the majority of the research on sonic seasoning has been conducted in Europe or the USA, typically in a within-participants experimental context. In the present study, we assessed the applicability of sonic seasoning in a large-scale between-participants setting in Asia. A sample of 1611 participants tasted one sample of chocolate while listening to a song that evoked a specific combination of cross-modal and emotional consequences. The results revealed that the music’s emotional character had a more prominent effect than its cross-modally corresponding attributes on the multisensory tasting experience. Participants expressed a higher buying intention for the chocolate and rated it as having a softer texture when listening to mainly positive (as compared to mainly negative) music. The chocolates were rated as having a more intense flavor amongst those participants listening to ‘softer’ as compared to ‘harder’ music. Therefore, the present study demonstrates that music is capable of triggering a combination of specific cross-modal and emotional effects in the multisensory tasting experience of a chocolate.

## 1. Introduction

Sonic seasoning refers to music or soundscapes that have been selected, or else deliberately produced, in order to trigger specific effects on the multisensory tasting experience [1,2,3,4]. The approach derives from the framework of research on the cross-modal correspondences, which points to the majority of people tending to share systematic associations between features, attributes, or dimensions of experiences across the senses [5]. In this context, think, for example, of associating the high pitch of a sound with small size, or high elevation [6,7]. Music is, in fact, a rich production process involving a mix of specific auditory elements that are combined during composition (e.g., frequency ranges, pitch, tempo and loudness, amongst many other attributes). Thus, there may be several auditory features/dimensions that can be associated with elements from a wide range of possible tastes and flavors in a customized fashion. Such multisensory customization potentially allows for the modulation or modification of the consumers’ experience of particular attributes of food and drink.

Musical tempo, for instance, has been shown to affect pleasure and arousal [8], while also affecting the tasting experience and shopping behavior [9]. Furthermore, the speed at which people eat and drink has also been shown to be affected by musical tempo [10,11]; cf. [12]. What is more, a number of studies have revealed that participants tend to drink more when the loudness of the background music goes up [13]. The perceived taste/flavor of alcoholic drinks can also be modified (either enhanced or diminished), when listening to background music at different loudness levels (cf. [14,15]; see also [16]).

Different studies have also shown that certain pitch or auditory frequency ranges correspond with specific taste/flavor sensations, such as bitterness, sourness, and sweetness [17,18,19,20]. Based on these insights, music can be produced or selected in order to deliberately correspond to specific taste sensations [1,18,21,22,23,24,25,26]. Using such an approach, Crisinel et al. demonstrated that the reported taste of samples of cinder toffee could be modulated by presenting a ‘sweet’ vs. ‘bitter’ background soundscape [1]. Meanwhile, Watson and Gunther [26] have suggested that the sound of the trombone is more strongly associated with bitterness than the sound of a clarinet. Reinoso Carvalho et al. [25] have also reported that what people listen to (one ‘sweet’, one ‘bitter’, and one in-between music clip) enhances the corresponding ratings of the taste of chocolate samples. Thus, not only can be music carefully selected, or soundscapes can correspond to particular taste/flavor attributes, but it is possible to influence such gustatory sensations when eating and/or drinking while experiencing congruent, as compared to incongruent, music [27,28].

The emotions that are associated with the music that happens to be playing in the background can also be transferred to, and thus influence, the tasting experience while eating and drinking [29,30,31,32]; see [33] for an overview of the literature on ‘sensation transference’. The scientific evidence that has been published to date is consistent with the view that such emotions tend to mediate similar cross-modal effects as those reported above; e.g., [29,30,31,32,34]. For example, Kantono et al. [29] reported that the perceived sweetness and bitterness of ice cream was modulated by whether the music playing in the background was liked or disliked, respectively. Reinoso-Carvalho et al.’s [31] research also revealed that the flavor of beer is rated differently as a function of whether people are listening to music that happens to trigger positive vs. negative emotions. Meanwhile, Ziv [32] reported that biscuits are rated as tasting better when participants listen to pleasant (as compared to unpleasant) music. Seo and Hummel [35] have also shown that the hedonic valence associated with what is heard can be transferred to olfaction, and that such a transference of valence is not necessarily correlated with how pleasantly people rate the relevant smells. The latter observation is important when considering the crucial role played by olfaction in the case of multisensory flavor perception [36,37,38].

In summary, based on the sonic seasoning research that has been published to date, cross-modally corresponding music, or music that is able to prime specific emotions, opens up the possibility of enhancing specific aspects of the multisensory tasting experience. However, the majority of the existing literature on this topic has been conducted in one specific type of population (European), and under somewhat rigid conditions (i.e., adopting a within-participants experimental design). Hence, the question of whether the same results would also be observed under more ecologically valid testing conditions remains to be further addressed. Moreover, if cross-modally corresponding music or music that is able to prime particular emotions can affect the tasting experience, one might wonder what would happen with music that triggers both effects in combination? To the best of our knowledge, the latter question has not yet been assessed empirically. In order to address these questions, we conducted a large-scale quantitative sonic seasoning assessment in an Asian country using a between-participants methodology.

### 1.1. Theoretical Framework

Within-participants experimental designs have been used in the majority of previous sonic seasoning research. In a typical study, different sounds are presented to the same listener, while experiencing a variety of food and/or drink items. For instance, Spence and his colleagues [4] noted that 15 out of 19 studies involving different associations between sound and taste/flavor were designed on a within-participant basis (see their Table 1). In parallel, such sounds, when presented to the same listener, are usually chosen in order to trigger contrasting effects (say, a pair of sounds that are able to elicit contrasting emotions, or perhaps another pair of cross-modally corresponding sounds where each tends to be associated with different gustatory sensations, such as sweet, sour, bitter, or sour).

The decision to present contrasting pairs of auditory stimuli to the same listener is usually inspired by the theory of cross-modal correspondences [5], according to which contrast (or more specifically, relative compatibility) is often crucial for the correspondences to be observed, and especially those involving the auditory parameter of pitch [39,40,41]. However, when looking for wider applicability, sonic seasoning should not constrain, for example, a consumer towards contrasting different sounds in order to be able to appreciate how the tasting experience can be affected by music. It is perhaps more feasible to foresee that sonic seasoning applied to the end consumer will involve just one sound cue being individually delivered (i.e., as an audio logo, and/or perhaps as a sonic jingle). Hence, as a first step, it was considered important to further establish whether these assessments are also relevant in a between-participants experimental setting (i.e., [42,43,44,45]).

Second, while looking for the experimental tasting stimuli, it was decided to work with chocolate, a somewhat challenging foodstuff, given the marked individual differences in liking for dark (i.e., less sweet) chocolate that have been reported in the literature [46].

Third, since most sonic seasoning studies have been conducted in Europe, the USA, or Australasia, we decided to conduct the present study in Asia (specifically, in South Korea). South Korea, as part of Southeast Asia, is one of the fastest growing markets in the world, especially with regards to technology [47,48]. Thus, this population may be framed as strategic when it comes to technological innovation in the sensory and consumer sciences.

Finally, as most of the existing evidence shows, music that is mainly produced or characterized with cross-modal correspondences or emotions in mind can affect the multisensory tasting experience [4,18]. Moreover, it has recently been suggested that the effects triggered by ‘emotional’ songs tend to more pronounced than those triggered by ‘cross-modally corresponding’ songs during within-participants sonic seasoning studies [2]. Nevertheless, our assumption here was that most music would likely trigger both effects.

### 1.2. The Present Study

The present study demonstrates that music capable of triggering a combination of specific cross-modal and emotional effects influenced the multisensory chocolate tasting experience in a predominantly South Korean population. Given the latest recommendations from the American Statistical Association to state overall evidence [49,50,51], our research was interpreted in terms of the robust Bayesian perspective.

We focused on two main research questions. First, we wanted to check whether two pairs of contrasting songs that were known to trigger specific cross-modal, or emotional effects would actually evoke a combination of these effects. As for the second question, we wanted to assess whether the effects prompted by the predominantly ‘emotional’ music would be more salient than those triggered by the ‘cross-modally corresponding’ music in a between-participants context (as recently suggested by [2]).

## 2. Materials and Methods

### 2.1. Experimental Design

In this study, the participants tasted a single piece of milk or dark chocolate while listening to one of four songs from two pairs of pre-existing primarily emotional or cross-modally corresponding music, depending on the condition to which they were randomly assigned during the experiment.

Prior to and after tasting the chocolate, the participants were instructed to clean their palates with tap water. After tasting the chocolate, they had to answer a survey related to their chocolate experience while listening to the music (“how much they liked the flavor of the chocolate”, “how much they thought the music matched the flavor of the chocolate”, the sweetness/bitterness/flavor-strength/texture of the chocolate, “how much they liked the music that they were listening to while tasting the chocolate”, their buying intention, and their willingness to pay (WTP) for the chocolate). They were also asked to provide basic demographic data (age and gender). Table 1 provides further details concerning the variables assessed in the survey implemented on the Qualtrics online platform. Importantly, the order of presentation of choices of answers was randomized.

### 2.2. Participants

A total of 1611 participants took part in this study (see Part 1 of [52] for the corresponding raw data). During sampling days, participants were selected by means of convenience sampling via offline crowdsourcing [53,54]. Further details of the sample and its sub-division across the experimental conditions is presented in Table 2. In order to verify the sample size, a power analysis was performed based on Friedman’s simplified determinations of statistical power; see Table 1 of [55]. Considering 95% confidence (α = 0.05), effect size of 0.15, and power between 0.8 and 0.9, the estimated sample size needed would be between 340–450 individuals for each between-participants experimental condition.

Sampling took place at the Asia Culture Center, located in Gwangju, Korea, between June and August in 2019. This was conducted during the ACT Festival as part of the Food Hack experience. The data was filtered in order to keep only participants between 15 and 80 years old.

### 2.3. Stimuli

Two types of chocolates were used: milk (Callebaut n. 823, milk-based with 33.6% minimum cacao solids), and dark (Callebaut n. 811, without-milk, with 54.5% minimum cacao solids). Each piece of chocolate had an approximate diameter of 1 cm, and weighed roughly 1 g.

Two pairs of songs were used as the two experimental musical conditions. The first pair of songs came from a recent study by Reinoso-Carvalho et al. [24]. Here, two contrasting songs were composed, considering mainly cross-modal correspondences between taste/flavor and music [5]. The bouba-kiki effect discussed by Köhler [56], and associations between touch and sound reported by Eitan and Rothschild [57], were used as a reference source for the musical composition process. Such cross-modally corresponding songs were produced in order for one to correspond with smoothness/softness (namely soft), and the other with roughness/hardness (namely hard). Both songs were approximately 1 min long; see [24] for corresponding songs and more details on this study).

The second pair of songs had been used previously in a study reported by Reinoso-Carvalho et al. [31], in which the music was shown to prompt contrasting emotional effects (either positive or negative). These songs were also approximately 1 min in duration. Clip 1 represented positive emotions, while Clip 4 represented negative emotions (see [31] for corresponding songs and further details). Note that this selection of music was also designed to try and avoid any semantic associations that may be primed by a particular style of music, as both songs resemble classical music (cf. [58,59,60]). Importantly, Clip 4 has a singing female voice, and the language of the singer may play a priming role in these types of studies. However, in this case, the song was being interpreted in a language that, to our belief, would not be understood by the majority of the listeners where this study was conducted.

In this study, these two pairs of songs were pre-tested for their cross-modal correspondences and emotions. An online survey was sent to 135 participants worldwide, where they had to rate the four songs in a within-participants design. Here, each participant listened to each of these songs, and rated each of them in terms of the cross-modal correspondences between music and taste/flavor in mind (sweetness, bitterness, hardness/roughness, softness/smoothness), and emotions (five positive—active, attentive, enthusiastic, excited, inspired; and five negative—distressed, irritable, nervous, scared, upset). All ratings were based on 5-point scales, with 1 associated with ‘not at all’, and 5 with ‘very much’. Each cross-modal rating was analyzed individually. The emotional ratings corresponded to a reduced version of the Positive and Negative Affect Schedule (PANAS), and were analyzed following this method (see [61,62] for details). The complete data analysis can be found in the Appendix A. Note that such data were collected for this study and not published in other studies. In brief, the emotional musical pair (positive and negative songs) triggered the most contrasting effects across both cross-modal and emotional ratings when compared to the cross-modally corresponding pair of musical stimuli (soft and hard songs).

### 2.4. Procedure

During the main test, participants were exposed to similar environmental conditions, which included comfortable temperature, regular/low background noise, and fairly stable lighting. All of the participants used the same type and model of tablets (Apple iPad), and the same type and model of headphones (Cresyn). The music playback volume was set in order to be similar in all systems (approximately 70 dB +/− 6 dB). From Tuesday to Sunday, between 10 am and 6 pm, visitors to the Food Hack festival who passed near to the experimental area were invited to join the study. It was explained that they would be taking part in a short study which involved tasting chocolate while listening to a musical clip. There were eight individual experimental booths open at all times. Participants were not able to hear each other. The participants were also reminded that they should take part in the experiment individually, and they were not able to participate more than once. During the experiment, each participant tasted the chocolate sample from a plate, and was instructed to let the chocolate melt in their mouth for most of the time (i.e., they were asked not to bite or chew it immediately). Prior to the tasting process, participants were advised to focus on the chocolate’s most salient taste/flavor attributes while listening to the music. The entire experimental task took no more than 5 min to complete.

### 2.5. Data Analysis

In the main test, the participants mostly reported their perception of the different characteristics (outcomes) of the experience on a scale from 1 to 100 (except for WTP, which was an open question). The current data were collected for this study, and showed strong non-symmetric behavior among the bounded scales, with large numbers of respondents selecting extreme values close to the boundaries, which contradicts the assumptions of traditional multivariate regression approaches to analysis (i.e., residuals cannot be normally distributed when responses are near/at the boundaries of the response space). While approximations through multivariate linear regressions are technically feasible, they would not be appropriate for data showing such features.

In order to overcome this problem, each outcome, j, for each individual, i (Y[i,j]), was mapped to the unit range (through rescaling of the original data to fit the (0,1) range), and assumed to follow a Bayesian, multi-response, multivariate, logit-normal distribution with outcome-specific intercepts and slopes, and common covariance structure across outcome measures [63]. The logit-normal distribution can take many standard shapes (including U-shapes and J-shapes, which are needed in this analysis), and is appropriate for bounded scores, offering the flexibility needed for responses near the boundaries of the support. Outcome-specific intercepts and slopes are needed since the association of each covariate with each of the outcomes/responses can be largely different, even if the outcomes/responses are correlated.

While modeling each outcome, j, independently was possible, joint modeling of all the outcomes provides enhanced estimation power due to the information borrowing resulting from the high correlation observable between some of the outcomes (both positive and negative correlation). Hence, the approach proposed in this manuscript is a multi-response version of the multivariate logit-normal regression model.

Bayesian approaches are flexible and adaptive to different sample sizes, especially where reliance on the central limit theorem can be questionable. Additionally, where available, they provide a natural form to incorporate any prior information available, either from prior studies or from expert opinion. For the purpose of this study, we assumed that there was no prior information available through non-informative priors for all parameters and hyperparameters in the models.

The set of covariates considered in this study include: (1) Negative/Hard, which is a binary measure for each individual, i, as the type of music; (2) DarkChoc, which represents a binary measure for whether the type of chocolate was dark; (3) Age, expressed as a continuous covariate; (4) Female, a binary measure for whether the declared gender was female; and (5) OtherGender, a binary measure for whether the declared gender was other. Note that reference categories for the results correspond to soft music, milk chocolate, and male respondents. All the associations are measured with respect to these categories in an additive form.

The multi-response, multivariate, logit-normal regression model takes the following form:

logit(Y[i,]) = α[1,] + α[2,] × Negative/Hard[i] + α[3,] × DarkChoc[i] + α[4,] × Age[i] + α[5,] × Female[i] + α[6,] × OtherGender[i] + ε[i].(1)

The term logit(Y[i,]) in Equation (1) represents the J-dimensional, component-wise logit transformation (logit(x) = log(x/(1 − x)) of the J-dimensional vector Y[i,] of outcomes, where Y[i,] ⊂ (0,1)^J^, while the transformed variable is logit(Y[i,]) ⊂ ( − ∞, ∞)^J^. The components α[k,j] in the 6 × J parameter matrix α can be interpreted as the expected change resulting from a unit increase in the corresponding covariate, k, on the odds of the untransformed outcome, (Y[i,]), for each of the five covariates, k = 2,..,6, outlined in Equation (1). The intercept vector α[1,] represents the mean logit-transformed response for each of the outcome variables.

Finally, the error term ε[i] ~ MVN_J_(0,Θ), which represents the features not captured by the data, follows a J-dimensional multivariate Gaussian density with mean vector, 0, and common J × J precision matrix, Θ, across individuals, i, which further allows for information borrowing across individuals and responses (ε follows a matrix-normal density with a block-design by individual).

The Bayesian form of the model is completed with independent, non-informative Gaussian priors for the intercept and slope matrix, α, and a non-informative diagonal Wishart prior for the precision matrix, Θ. In cases where information is available to inform those priors, this information can be easily incorporated. However, for the purpose of this study, all priors are set as non-informative.

The quantities of interest (see Section 2 in Table 1) are the directionalities of each of the outcomes measured in this study across music influences (Negative/Hard), namely the directionalities (whether positive, negative, or negligible) of the components in the J-dimensional vector, α[2,], after accounting for the other covariates and an overall mean response vector. The model output represents the full (joint) posterior distribution of the different parameters, and we evaluate the associations through an assessment of the mass of the marginal distributions in the Results section (again, whether positive or negative).

The second type of model, which measures the association between the covariates and the WTP variable (see Section 3 in Table 1) was assessed using a Bayesian Poisson multivariate regression. Willingness to pay (single outcome per individual) was expressed as a positive, integer-based value, Z[i] ⊂ [0, ∞), representing how much the respondent was willing to pay for the choice offered given the characteristics (both sociodemographic and environmental). This value was modeled through a Poisson multivariate regression model with the following form:

Z[i]~Poi(λ[i]), where

Log(λ[i]) = β[1] + β[2] × Negative/Hard[i] + β[3] × DarkChoc[i] + β[4] × Age[i] + β[5] × Female[i] + β[6] × OtherGender[i].(2)

The set of covariates in Equation (2) is the same as the one used in the previous model, and the reference categories also remain unchanged. Poi(λ[i]) represents a Poisson distribution with an individual-specific latent mean parameter, λ[i], and the log-transformed mean, log(λ[i]), is related to the covariates linearly through the parameter vector, β.

The 6-dimensional parameter vector, β, can be interpreted as follows: for every unit change in the covariate, we anticipate an exp (β) change in the expected value of Z[i]. For example, the use of a Negative/Hard music experience is associated with an additional exp (β[2]) multiplicative effect in the expected willingness to pay. For example, if exp(β[2]) = 1.40, then the Negative/Hard music is associated with a 40% increase in willingness to pay, while if exp(β[2]) = 0.80, then the Negative/Hard music is associated with a 20% decrease in willingness to pay (always compared to the reference category, while holding all other covariates constant). Across parameters, for values of β < 0, the willingness to pay is reduced under larger values of the covariate (or when the binary variable is present), while the opposite occurs for values of β > 0. Posterior distributions for β are reported in the Results Section.

In a similar fashion to the previous model, the analysis can be further enhanced if there is prior information available, though in the case of our study, the Bayesian model proposed is completed with non-informative Gaussian priors for the β vector.

All models were coded and fitted using OpenBUGS version 3.2.3 [64].

## 3. Results

### 3.1. Interpretation of Results

Table 3 and Table 4 summarize results of the Bayesian multivariable multivariate logit-normal regression model, where the parameters are denoted by α. Table 3 summarizes the results regarding the associations prompted by the music primarily chosen due to its cross-modal features on the evaluation of the chocolate. Table 4 summarizes the results concerning associations brought by the music primarily chosen due to its emotional characteristics in the same context (in both cases, excluding WTP ratings). Table 5 summarizes the results of WTP for both musical conditions. The latter analysis is based on a Bayesian Poisson regression model, and the parameters are denoted by β.

Intercepts are denoted as α[1,]. α[2,] represents associations between music and chocolate ratings. α[3,] and α[4,] capture the effects of type of chocolate (difference between dark and milk) and age (unit per additional year of age), respectively. α[5,] and α[6,] are the effects of gender (α[5,] = differences between female and male; α6 = differences between ‘other’ and male). The aforementioned also applies to the parameter vector β.

All parameters indicate the ‘additional’ impact of moving away from a reference category (i.e., the difference between Y and X). For example, regarding α[2,], Y represents the Negative/Hard song, while X represents the Positive/Soft one. Hence, if we take one α[2,] variable as an example in Table 3 (say, the variable flavor liking rating), the parameter represents the estimated non-linear impact of the differences between the hard and soft songs on the flavor liking ratings for the chocolate. Moreover, the same example in Table 4 would be the impact of the differences between the negative and positive songs on flavor liking ratings for the chocolate, and so on. All of these results are estimated upon accounting for all the other covariates, and incorporating the correlations among all the existing dependent variables. Note that results related to α[6,] are not interpreted here, since the sample size associated with those participants who declared themselves as being ‘other’ gender was too low (and the corresponding posterior density too wide), as compared to those who declared being male or female participants.

In a similar fashion, the interpretation for β is that Y brings a (exp(β) − 1) × 100% higher willingness to pay than X. Again, with regards to β[2], Y represents Negative/Hard, and X Positive/Soft songs, depending on musical condition.

Three levels of probabilistic strength on the evidence were considered (strong evidence, weak evidence, or no evidence of positive/negative associations). Negative associations suggest a more salient effect of X as compared to Y on the specific rating of the multisensory tasting experience, and vice-versa. We report strong evidence of associations where the 95% Bayesian posterior credible interval fell within the positive or negative numerical domain. The 95% credible intervals where most of the mass was within the positive or negative numerical domain were considered as prompting weak evidence of associations. Finally, no evidence is reported within a 95% credible interval with bounds in both the positive and negative domains, along with high probabilities in both the positive and negative domains.

### 3.2. Tasting Chocolate While Listening to Music Primarily Chosen Due to its Cross-Modal Features (Soft vs. Hard)

The parameter values corresponding to flavor liking, buying intention, song liking, flavor–music matching, flavor intensity, and softness of the chocolate’s texture demonstrated strong evidence of negative associations with this music. This suggests that the flavor of the chocolate was preferred by participants, and their buying intention was higher, when tasting it alongside soft music as compared to those participants who tasted it while listening to hard music. Similarly, these results also suggest that the soft music was liked more, and it was considered as a better match for the taste/flavor of the chocolate as compared to the hard music. Participants listening to soft music also rated the chocolate as having a more intense flavor and a softer texture when compared to those participants who tasted the chocolate while listening to the hard music instead. There was no evidence that participants rated the sweetness, bitterness, sourness, and hardness (as in texture) of the chocolate differently as a function of which of these two songs they listened to (see α[2,] results in Table 3).

The α[3,] results in Table 3 highlight the estimated effects of the type of chocolate on ratings. In particular, ratings related to flavor liking, sweetness, softness, and buying intention revealed strong evidence of negative associations with the type of chocolate. Ratings of bitterness, sourness, and hardness showed strong evidence of positive associations with type of chocolate. Ratings related to flavor–music matching, flavor intensity, and song liking did not highlight any associations. Taken together, these results suggest that the flavor of milk chocolate was preferred, and regarded as sweeter, less bitter/sour in terms of flavor, softer/less hard, and as triggering higher buying intention as compared to the dark chocolate, and regardless of the music playing in the background. Age-related parameter values revealed strong evidence of associations between age and flavor liking, sweetness, and sourness ratings (see α[4,] in Table 3). Gender did not seem to follow any associative patterns in this musical condition (see α[5,] and α[6,] in Table 3).

The results related to WTP for the chocolates in this condition indicated strong evidence of negative associations with these two songs. This would suggest that the participants were willing to pay more for the same chocolate while listening to the soft as compared to the hard music. Additionally, all of these ratings were strongly associated with the type of chocolate, age, and gender (see Table 5 for all WTP results).

In summary, there was overall evidence suggesting that participants rated the flavor of both chocolates as more intense while listening to soft music when compared to the effects prompted by listening to hard music. Moreover, soft music was generally preferred, and thought of as a better match for the flavor of both types of chocolates compared to the hard music. The rest of the results cannot be directly associated to the effects of the different songs on the multisensory tasting experience, as they showed a strong effect of the covariates, and especially the type of chocolate.

### 3.3. Tasting Chocolate While Listening to Music Mainly Chosen Due to its Emotional Characteristics (Positive vs. Negative)

Parameter values corresponding to buying intention, music liking, flavor–music matching, sweetness, and texture softness/hardness ratings leaned towards strong negative associations with emotional music. Similarly, ratings related to flavor liking revealed a weak negative association with emotional music. Hence, these results suggest that buying intention for the chocolate was higher when tasting it alongside positive music as compared to when tasting it while listening to negative music instead. Moreover, positive music was preferred, and it was considered as a better match for the flavor of the chocolate when compared to the response elicited by the negative music. The participants also rated the chocolate as tasting sweeter and as having a softer/less hard texture when it was evaluated with positive music compared to those who were listening to negative music during the evaluation of the chocolate. The results also suggest that those participants listening to positive music may have preferred the flavor of the chocolate compared to those listening to negative music. There was no evidence to suggest that participants rated bitterness, sourness, and flavor intensity of the chocolate differently based on the music to which they were listening (see α[2,] results in Table 4).

The α[3,] results in Table 4 report the estimated effects of the type of chocolate. Parameter values corresponding to ratings related to flavor liking and sweetness showed strong evidence of negative associations with type of chocolate, while values related to ratings of bitterness, sourness, and flavor intensity showed strong evidence of positive associations with type of chocolate. Parameter values corresponding to ratings related to flavor–music matching, texture softness/hardness, song liking, and buying intention did not show associations with the type of chocolate. The latter suggests that the taste/flavor of the milk chocolate was preferred, regarded as sweeter, and less bitter/sour/intense in terms of its taste/flavor when compared to the dark chocolate, and regardless of the music that happened to be playing in the background. Age-related parameter values demonstrated strong evidence of an association between age and flavor liking, sweetness, and sourness ratings (see α[4,] in Table 4). Gender did not follow any associative patterns in this musical condition (see α[5,] and α[6,] in Table 4).

The results related to the WTP for the two types of chocolate in this emotional music condition revealed strong evidence of a negative association in terms of WTP. This would suggest that participants were willing to pay more for the same chocolate while under the influence of the positive music as compared to the negative music. Additionally, all of these ratings were strongly associated with the type of chocolate, age, and gender (see Table 5 for all WTP results).

In brief, these results suggested that participants were rating both chocolates as having a softer/less-hard texture under the multisensory effects of the positive music as compared to the negative music. The buying intention for the chocolate also seemed to be generally higher under the influence of the positive music as compared to the negative music. Moreover, the positive music was generally preferred and thought of as a better match for the taste/flavor of both chocolates when compared to the negative music.

**Figure 1:**
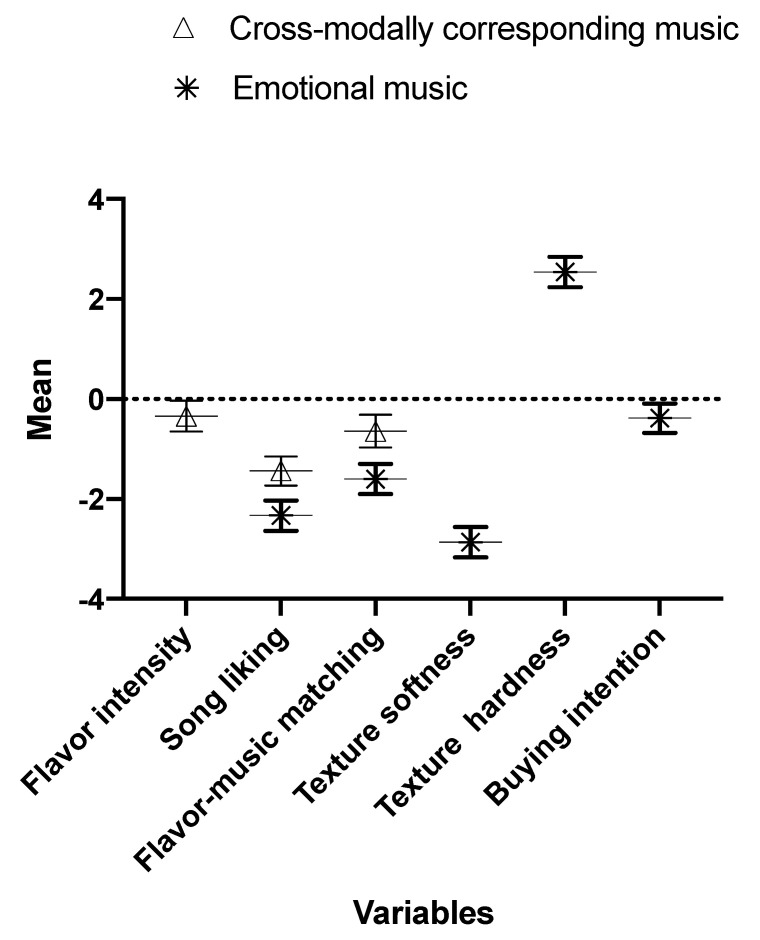
Summary of results where there is strong evidence that the associations being reported for each variable were not (solely) driven by the covariates, but are attributed to the effects triggered by the music while tasting the chocolates (except for WTP, since it was calculated via a different model). The results related to the music primarily chosen due to its cross-modal features are those where the posterior mean is being represented with a triangle. The results related to the music mainly chosen due to its emotional characteristics are those where the posterior mean is represented by an asterisk. Bars represent the upper and lower limit of the corresponding 95% credible interval.

Figure 1 summarizes the results where there was strong evidence of associations attributed to the effects that listening to the different music was triggering during the multisensory experience of the participants while tasting the chocolates (except for WTP, since it was calculated via a different model).

## 4. Discussion

In the present study, a large-scale sonic seasoning between-participants experiment was conducted in an Asian country (South Korea). Here, the effects of combined cross-modal correspondences and emotions elicited by the same song on chocolate tasting were observed. First, we verified whether two pairs of contrasting songs (varying in terms of their cross-modal associations and emotions) would evoke the expected effects on participants while being tested in such between-participants setting. Second, we assessed whether the effects prompted by the music mainly chosen due to its emotional characteristics would continue to be more prominent than those triggered by the music primarily chosen due to its cross-modal features, as it has been argued recently [2].

In general, both tests revealed the expected effects. These new findings are therefore relevant for the field since, for the first time, the effects of blending cross-modality and emotions in sonic seasoning are being reported. In this way, we add crucial information to support a greater applicability of sonic seasoning in a range of different contexts. Here, the novelty that the obtained results were analyzed using what is a rather robust, Bayesian statistical approach, may also be of importance for some researchers.

### 4.1. The Effects of Blending Cross-Modality and Emotions in Sonic Seasoning

The evidence obtained in the present study indicates that music produced or selected with sonic seasoning in mind can still trigger some of the expected effects in a multisensory tasting experience under the testing conditions implemented here (see α[2,] results in Table 3 and Table 4). Moreover, the overall evidence points towards music mainly chosen due to its emotional characteristics as triggering more prominent effects on the multisensory chocolate tasting experience of the participants when compared to those effects elicited by the music that had primarily been chosen for its cross-modal correspondence with specific taste/flavor features (see Figure 1).

**Figure A1:**
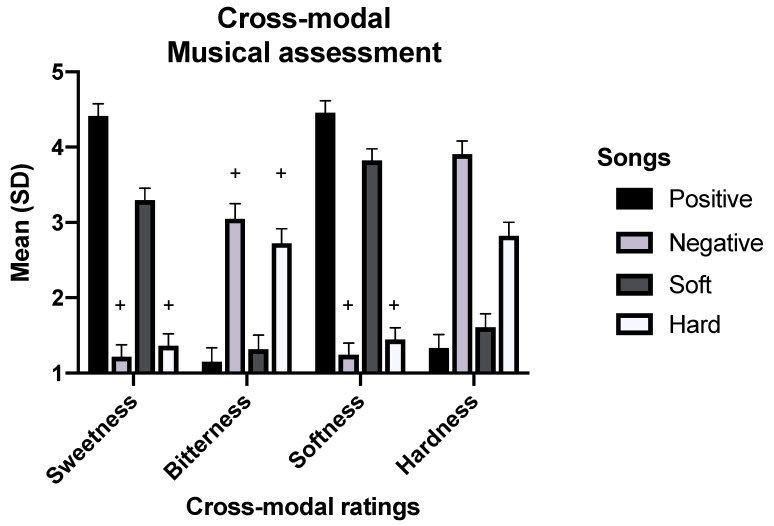
Summary of cross-modal ratings during the pre-test of the songs. Bars represent the mean values with corresponding standard deviations (SD). Plus signs show the only pairs of bars where there was no significant difference between such means at 95% confidence.

**Figure A2:**
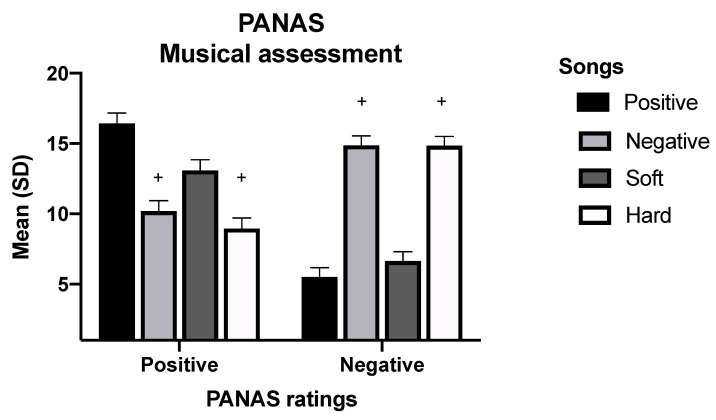
Summary of the Positive and Negative Affect Schedule (PANAS) ratings during the pre-test of the songs. Bars represent the mean values with corresponding standard deviations (SD). Plus signs show the only pairs of bars where there was no significant difference between such means at 95% confidence.

As the pre-test initially suggested, the participants rated the emotional musical pair as evoking more contrasting emotional and cross-modal ratings when compared to the cross-modally corresponding musical pair (see Figure A1 and Figure A2 in the Appendix A). In the main test, for the emotional musical condition, there was evidence to suggest that participants were rating the chocolate as softer while listening to positive music as compared to negative music. In addition, the evidence also suggests that participants rated chocolates as having a harder texture when accompanied by negative music rather than positive music. In general, buying intentions for the chocolates were also shown to be higher under the influence of positive as compared to negative music. For the cross-modally corresponding musical condition, however, there was only evidence to suggest that the participants were rating either chocolate as having a more intense flavor under the influence of the soft music as compared to those effects elicited by the hard music. Therefore, it would appear that choosing music associated with particular emotions may provide a more robust means of promoting sonic seasoning effects under similar conditions to those explored in this study.

From a theoretical standpoint, it could be hypothesized that the emotional response triggered by music may be a more straightforward mediator during sonic seasoning, at least when compared to the effects elicited by cross-modally corresponding music. In fact, it has been argued that emotions mediate cross-modal correspondences between what we listen to, and taste, together with, for example, cross-modal congruence [31]. Furthermore, previous research has also suggested that the emotions elicited by music also seem to be less affected by cultural differences than sonic seasoning [2]. In summary, the path of primarily choosing music based on the emotions that can be evoked in the listener may be more optimal while designing meaningful sonic seasoning experiences as part of multisensory food experience design strategies. After setting the intended emotional musical scope, looking for cross-modality between what we listen to and what we taste might be more effective than doing the inverse (i.e., first setting a scope for cross-modal congruence, and then moving towards emotional enhancement of the tasting experience).

Note that in this study, the perceived texture of the chocolates was not measured using specific food science methods (e.g., texture profile analysis). Since some of the obtained results are related to the way music can affect the way consumers rate a chocolate’s texture (i.e., hardness, softness), future studies could replicate these findings while using some of these methods.

### 4.2. Towards Greater Applicability of Sonic Seasoning

The evidence reported here suggests that music can still modulate the perceived texture as well as the buying intention for different chocolates in less rigid experimental contexts (e.g., between-participants). Music that is produced or selected to be primarily cross-modally corresponding with specific gustatory sensations would appear not to be as effective as music that has primarily been selected due to its emotional features (cf. [2]). However, this methodology also shows that such music can still trigger specific effects on the multisensory tasting experience of chocolate, such as modulating the perceived flavor intensity (see Table 3). By conducting this study in Asia, we also provide further support that these results can be also be implemented in different cultural contexts (cf. [2]).

### 4.3. Sonic Seasoning: A Bayesian Approach

The results obtained in the present study suggest that the methodological decision to approach the strongly asymmetric behavior of the data analyzed in the main test of this study via logit-normal regression statistical models was effective. Bounded responses presenting such characteristics can be more effectively modeled through logit-normal regression models [63,65]. They represent an intuitive alternative to, for example, traditional multivariate approaches with near-the-boundary outcomes, like those presented in this and other similar studies [65]. In fact, the number of categories in integer-based scales, and corresponding parameter space, such as those present in this study, would be prohibitive and impractical across alternatives viable for integer-based fine scales, such as those stemming from categorical data models (e.g., [2]). In summary, the proposed Bayesian framework naturally allows for interpretations based on full representation of the existing evidence (e.g., weak, strong, or no evidence), and aligns with recent recommendations within the statistical community (e.g., [49,50,51]).

### 4.4. Final Thoughts from the Practitioner Perspective

This report adds value to the corresponding literature while providing a setting for greater applicability of sonic seasoning across more diverse situations of multisensory food experience design. For instance, brands can use these findings as a baseline to produce music that may be able to enhance the multisensory tasting experience of their customers. Such music can be used as part of promotion, as in advertising and/or experiential strategies (e.g., [66]). When considering the further adoption of individual-targeted marketing, not only a particular set of products can be marketed based in past purchasing experiences, but sonic seasoning may also be useful when designing experiences to match the products as well as the customer’s characteristics (e.g., based on personal musical preferences). In fact, multisensory experiences involving music and food/beverage consumption seem to be increasingly appealing to those working in this sector of product and experience design (i.e., see Campari [67], Godiva [68], Jägermeister [69], Tramontina [70]). 

Compared to previous similar studies, it is now clearer that such ideas can be implemented in less rigid settings without, for example, the need to incorporate within-participants contrast (cf., [1,2,24]). This makes it easier (i.e., more flexible) to consider sonic seasoning as adding value during the design of food experiences for the end consumer. Here, we could think of sonic seasoning applied to the end consumer as one sound cue being individually delivered (i.e., as an audio logo, and/or perhaps as a sonic jingle as part of a food/drink promotion’s campaign). Importantly, the user-friendly way in which the experimental task was designed in this study allows for further adoption of different ways of sourcing for sonic seasoning, including potential online options (i.e., e-commerce). This is mainly due to the fact that the experimental conditions to be controlled while sourcing for sonic seasoning have been significantly optimized in this assessment, and shown to be effective. Hence, online sonic seasoning sampling (something that, to our knowledge, has not yet being implemented) may become more feasible as long as the end user has access to the specific food/beverage at stake, accompanied by some basic gear such as a graphics user interface (GUI), headphones, and internet. 

## Figures and Tables

**Table 1:** Experimental design, including the variables that were sampled, and the measures used in order to assess each variable. Note that the presentation of variables was randomized.

Section of Questionnaire	Variable	Measurement System
Demographics	Age	Open numerical
	Gender	1 = male; 2 = female; 3 = other
2. Rating scales	Flavor liking	100-point rating scales (1 being ‘not at all’; 100 being ‘very much’)
	Flavor–music matching	
	Chocolate sweetness	
	Chocolate bitterness	
	Chocolate sourness	
	Song liking	
	Flavor intensity	
	Buying intention	
	Texture hardness	
	Texture softness	
Open question	Willingness to pay (WTP)	Numerical, which included a message with local reference

**Table 2:** Sample sizes (n), average age with corresponding standard deviation (SD) in years, as well as gender ratio, for each between-participants experimental condition.

n_Positive_ _Emotional Song_ Mean Age (SD) Gender Ratio	n_Negative_ _Emotional Song_ Mean Age (SD) Gender Ratio	n_Cross-Modally Corresponding Music_ _Smooth Song_ Mean Age (SD) Gender Ratio	n_Cross-Modally Corresponding Music_ _Hard Song_ Mean Age (SD) Gender Ratio	n_Total_ Mean Age (SD) Gender Ratio
376 30.1 (10) 65% females	450 30.8 (11) 68% females	365 30.7 (11) 63% females	420 30.5 (12) 64% females	1611 30.5 (11) 65% females

**Table 3:** Posterior means, posterior standard deviations, 95% credible intervals, and posterior medians for all parameters in the Bayesian logit-normal cross-modally corresponding music regression model. Values highlighted in bold represent stronger evidence of associations. Values in italics constitute weaker evidence of associations. All other values represent no evidence of associations.

	Variable	Mean	SD	95% Credible Interval	Median		Variable	Mean	SD	95% Credible Interval	Median
α[1,] Intercept	**Flavor liking**	**2.197**	**0.283**	**(1.638, 2.752)**	**2.198**	α[4,] Age	**Flavor liking**	**−0.019**	**0.007**	**(−0.033, −0.005)**	**−0.019**
	**Flavor–music match**	**−1.106**	**0.273**	**(−1.642, −0.570)**	**−1.106**		Flavor–music match	0.011	0.007	(−0.003, 0.024)	0.011
	**Sweetness**	**2.621**	**0.278**	**(2.081, 3.166)**	**2.620**		**Sweetness**	**−0.024**	**0.007**	**(−0.038, −0.010)**	**−0.024**
	**Bitterness**	**−3.098**	**0.274**	**(−3.64, −2.559)**	**−3.097**		Bitterness	0.007	0.007	(−0.006, 0.021)	0.007
	**Sourness**	**−3.639**	**0.272**	**(−4.179, −3.106)**	**−3.637**		**Sourness**	**0.020**	**0.007**	**(0.007, 0.034)**	**0.020**
	*Flavor intensity*	*−0.313*	*0.275*	*(−0.849, 0.224)*	*−0.313*		Flavor Intensity	−0.005	0.007	(−0.019, 0.008)	−0.005
	**Texture softness**	**1.745**	**0.272**	**(1.216, 2.279)**	**1.744**		Texture softness	0.004	0.007	(−0.009, 0.017)	0.004
	**Texture hardness**	**−1.828**	**0.266**	**(−2.351, −1.303)**	**−1.828**		Texture hardness	−0.010	0.007	(−0.023, 0.003)	−0.010
	*Song liking*	*−0.479*	*0.262*	*(−0.986, 0.039)*	*−0.481*		Song liking	0.003	0.007	(−0.010, 0.015)	0.003
	**Buying intention**	**−0.936**	**0.281**	**(−1.485, −0.385)**	**−0.937**		Buying intention	−0.001	0.007	(−0.014, 0.013)	−0.001
α[2,] Music	**Flavor liking**	**−0.472**	**0.163**	**(−0.791, −0.151)**	**−0.473**	α[5,] Gender (Male/Female)	Flavor liking	−0.047	0.168	(−0.375, 0.282)	−0.048
	**Flavor–music match**	**−0.897**	**0.154**	**(−1.199, −0.594)**	**−0.898**		Flavor–music match	0.036	0.159	(−0.275, 0.348)	0.036
	Sweetness	−0.237	0.160	(−0.550, 0.076)	−0.237		Sweetness	0.050	0.165	(−0.273, 0.374)	0.049
	Bitterness	0.137	0.156	(−0.169, 0.442)	0.137		Bitterness	0.114	0.162	(−0.203, 0.431)	0.114
	Sourness	0.036	0.154	(−0.265, 0.338)	0.035		Sourness	0.028	0.159	(−0.284, 0.339)	0.027
	**Flavor intensity**	**−0.344**	**0.157**	**(−0.651, −0.036)**	**−0.345**		Flavor intensity	−0.071	0.162	(−0.389, 0.246)	−0.070
	**Texture softness**	**−0.360**	**0.154**	**(−0.662, −0.059)**	**−0.360**		Texture softness	−0.154	0.158	(−0.465, 0.155)	−0.154
	Texture hardness	−0.037	0.152	(−0.335, 0.262)	−0.037		Texture hardness	0.071	0.158	(−0.239, 0.377)	0.071
	**Song liking**	**−1.438**	**0.149**	**(−1.732, −1.148)**	**−1.438**		Song liking	0.017	0.152	(−0.283, 0.312)	0.017
	**Buying intention**	**−0.313**	**0.158**	**(−0.625, −0.003)**	**−0.314**		Buying intention	−0.096	0.165	(−0.419, 0.226)	−0.096
α[3,] Type of chocolate	**Flavor liking**	**−0.641**	**0.166**	**(−0.968, −0.316)**	**−0.641**	α[6,] Gender (Male/Other)	*Flavor liking*	*−0.403*	*0.681*	*(−1.738, 0.933)*	*−0.401*
	Flavor–music match	−0.047	0.158	(−0.357, 0.262)	−0.047		Flavor–music match	0.042	0.649	(−1.234, 1.309)	0.043
	**Sweetness**	**−1.314**	**0.163**	**(−1.634, −0.994)**	**−1.313**		*Sweetness*	*−0.863*	*0.672*	*(−2.180, 0.459)*	*−0.863*
	**Bitterness**	**2.229**	**0.159**	**(1.917, 2.542)**	**2.229**		Bitterness	0.176	0.659	(−1.116, 1.464)	0.180
	**Sourness**	**0.563**	**0.157**	**(0.255, 0.872)**	**0.564**		*Sourness*	*0.330*	*0.646*	*(−0.938, 1.593)*	*0.332*
	Flavor intensity	0.187	0.160	(−0.126, 0.499)	0.187		*Flavor intensity*	*0.278*	*0.658*	*(−1.018, 1.563)*	*0.279*
	**Texture softness**	**−1.582**	**0.157**	**(−1.890, −1.274)**	**−1.581**		*Texture softness*	*−0.292*	*0.644*	*(−1.546, 0.973)*	*−0.292*
	**Texture hardness**	**1.813**	**0.155**	**(1.508, 2.115)**	**1.813**		*Texture hardness*	*−0.801*	*0.642*	*(−2.059, 0.467)*	*−0.801*
	Song liking	0.185	0.151	(−0.110, 0.481)	0.185		*Song liking*	*0.409*	*0.626*	*(−0.818, 1.636)*	*0.409*
	**Buying intention**	**−0.497**	**0.161**	**(−0.814, −0.181)**	**−0.498**		Buying intention	−0.099	0.669	(−1.409, 1.211)	−0.103

**Table 4:** Posterior means, posterior standard deviations, 95% credible intervals, and posterior medians for all parameters in the Bayesian logit-normal emotional music regression model. Values highlighted in bold represent stronger evidence of associations. Values in italics constitute weaker evidence of associations. All other values represent no evidence of associations.

	Variable	Mean	SD	95% Credible Interval	Median		Variable	Mean	SD	95% Credible Interval	Median
α[1,] Intercept	**Flavor liking**	**2.245**	**0.297**	**(1.663, 2.823)**	**2.245**	α[4,] Age	**Flavor liking**	**−0.026**	**0.007**	**(−0.040, −0.013)**	**−0.026**
	*Flavor–music match*	*0.380*	*0.306*	*(−0.220, 0.981)*	*0.377*		Flavor–music match	0.000	0.007	(−0.014, 0.014)	0.000
	**Sweetness**	**3.049**	**0.280**	**(2.504, 3.598)**	**3.048**		**Sweetness**	**−0.032**	**0.007**	**(−0.045, −0.019)**	**−0.032**
	**Bitterness**	**−2.449**	**0.290**	**(−3.017, −1.878)**	**−2.449**		*Bitterness*	*−0.014*	*0.007*	*(−0.027, 0.000)*	*−0.013*
	**Sourness**	**−2.998**	**0.286**	**(−3.555, −2.435)**	**−2.999**		Sourness	0.001	0.007	(−0.012, 0.014)	0.001
	Flavor intensity	0.003	0.286	(−0.561, 0.553)	0.006		**Flavor intensity**	**−0.025**	**0.007**	**(−0.038, −0.012)**	**−0.025**
	**Texture softness**	**1.242**	**0.306**	**(0.647, 1.843)**	**1.242**		Texture softness	−0.006	0.007	(−0.020, 0.008)	−0.006
	**Texture hardness**	**−0.834**	**0.301**	**(−1.420, −0.243)**	**−0.836**		Texture hardness	−0.010	0.007	(−0.023, 0.004)	−0.010
	**Song liking**	**1.348**	**0.303**	**(0.757, 1.946)**	**1.345**		Song liking	−0.005	0.007	(−0.019, 0.009)	−0.005
	**Buying intention**	**−0.635**	**0.288**	**(−1.203, −0.077)**	**−0.634**		Buying intention	−0.010	0.007	(−0.023, 0.003)	−0.010
α[2,] Music	*Flavor liking*	*−0.250*	*0.152*	*(−0.548, 0.049)*	*−0.250*	α[5,] Gender (Male/Female)	*Flavor liking*	*0.261*	*0.163*	*(−0.060, 0.581)*	*0.261*
	**Flavor–music match**	**−1.602**	**0.155**	**(−1.905, −1.297)**	**−1.601**		Flavor–music match	0.140	0.166	(−0.185, 0.467)	0.140
	**Sweetness**	**−0.440**	**0.144**	**(−0.721, −0.158)**	**−0.439**		Sweetness	0.108	0.153	(−0.192, 0.408)	0.108
	Bitterness	0.232	0.147	(−0.056, 0.520)	0.232		Bitterness	0.079	0.157	(−0.227, 0.386)	0.079
	Sourness	0.006	0.148	(−0.282, 0.295)	0.006		Sourness	−0.175	0.157	(−0.487, 0.132)	−0.174
	Flavor intensity	0.150	0.145	(−0.132, 0.434)	0.149		Flavor intensity	−0.012	0.155	(−0.314, 0.294)	−0.011
	**Texture softness**	**−2.866**	**0.156**	**(−3.173, −2.562)**	**−2.866**		Texture softness	−0.051	0.167	(−0.380, 0.273)	−0.051
	**Texture hardness**	**2.541**	**0.154**	**(2.239, 2.844)**	**2.540**		Texture hardness	−0.218	0.166	(−0.542, 0.107)	−0.217
	**Song liking**	**−2.333**	**0.156**	**(−2.639, −2.028)**	**−2.333**		Song liking	0.029	0.165	(−0.294, 0.355)	0.029
	**Buying intention**	**−0.384**	**0.150**	**(−0.679, −0.090)**	**−0.383**		Buying intention	0.094	0.160	(−0.218, 0.408)	0.094
α[3,] Type of chocolate	**Flavor liking**	**−0.689**	**0.154**	**(−0.990, −0.387)**	**−0.690**	α[6,] Gender (Male/Other)	Flavor liking	−0.038	0.519	(−1.058, 0.974)	−0.037
	Flavor–music match	−0.048	0.159	(−0.358, 0.264)	−0.047		*Flavor–music match*	*−0.457*	*0.529*	*(−1.496, 0.583)*	*−0.457*
	**Sweetness**	**−1.488**	**0.145**	**(−1.773, −1.202)**	**−1.487**		*Sweetness*	*0.357*	*0.488*	*(−0.606, 1.315)*	*0.356*
	**Bitterness**	**2.214**	**0.150**	**(1.922, 2.508)**	**2.213**		*Bitterness*	*0.665*	*0.502*	*(−0.324, 1.645)*	*0.666*
	**Sourness**	**0.946**	**0.150**	**(0.653, 1.239)**	**0.945**		Sourness	−0.048	0.504	(−1.031, 0.950)	−0.047
	**Flavor intensity**	**0.522**	**0.148**	**(0.231, 0.812)**	**0.523**		*Flavor intensity*	*0.899*	*0.494*	*(−0.069, 1.866)*	*0.897*
	Texture softness	0.095	0.159	(−0.216, 0.405)	0.095		*Texture softness*	*0.518*	*0.532*	*(−0.526, 1.562)*	*0.518*
	Texture hardness	0.107	0.157	(−0.199, 0.416)	0.107		*Texture hardness*	*−0.966*	*0.528*	*(−2.001, 0.065)*	*−0.967*
	Song liking	−0.098	0.158	(−0.408, 0.210)	−0.097		*Song liking*	*−0.256*	*0.531*	*(−1.299, 0.785)*	*−0.254*
	Buying intention	−0.265	0.152	(−0.564, 0.035)	−0.266		*Buying intention*	*−0.701*	*0.512*	*(−1.703, 0.306)*	*−0.701*

**Table 5:** Posterior means, posterior standard deviations, 95% credible intervals, and posterior medians for all parameters in the Poisson cross-modally corresponding (left) and emotional (right) music regression models for willingness to pay (WTP) ratings. Values highlighted in bold represent stronger evidence of associations. Values in italics constitute weaker evidence of associations.

Cross-Modal					Emotional				
Parameter	Mean	SD	95% Posterior Interval	Median	Parameter	Mean	SD	95% Posterior Interval	Median
β[1]—Intercept	**6.626**	**0.005**	**(6.616, 6.636)**	**6.626**	β[1]—Intercept	**6.922**	**0.005**	**(6.913, 6.932)**	**6.922**
β[2]—Music	**−0.404**	**0.003**	**(−0.409, −0.399)**	**−0.404**	β[2]—Music	**−0.010**	**0.002**	**(−0.015, −0.005)**	**−0.010**
β[3]—Type of Chocolate	**0.313**	**0.003**	**(0.307, 0.318)**	**0.313**	β[3]—Type of Chocolate	**−0.162**	**0.002**	**(−0.166, −0.157)**	**−0.162**
β[4]—Age	**−0.008**	**0.000**	**(−0.008, −0.008)**	**−0.008**	β[4]—Age	**−0.006**	**0.000**	**(−0.007, −0.006)**	**−0.006**
β[5]—Gender (Male/Female)	**0.347**	**0.003**	**(0.341, 0.353)**	**0.347**	β[5]—Gender (Male/Female)	**0.197**	**0.003**	**(0.192, 0.202)**	**0.197**
β[6]—Gender (Male/Other)	**0.357**	**0.011**	**(0.335, 0.380)**	**0.357**	β[6]—Gender (Male/Other)	**−0.300**	**0.010**	**(−0.32, −0.281)**	**−0.300**

## Appendix A

### Data Analysis of the Pre-Test

The data of this pre-test was processed via multivariate analyses using SPSS 26. One analysis was conducted for the cross-modal ratings, and another for the emotional ones. In both cases, the ratings were declared as dependent variables, and the songs as fixed factors. Tukey was applied as a correction for the post-hoc analyses.

In general, a main effect of music was found for the cross-modal ratings [F(15, 1602) = 48.80, *p* ≤ 0.001, partial eta squared = 0.314]. Multiple comparisons revealed that most differences in ratings were significant (*p* ≤ 0.005). None of the differences were significant between participants in terms of the softness and Negative/Hard songs (*p* = 0.075), sweetness between Negative/Hard songs (*p* = 0.196), and bitterness between Sweet/Soft songs (*p* = 0.208). The positive song evoked the sweetest (M_sweet_ = 4.41, SD = 0.81), and softest ratings (M_soft_ = 4.46, SD = 0.84), followed by the soft song (M_sweet_ = 3.30, SD = 1.29; M_soft_ = 3.82, SD = 1.24). Hard (M_sweet_ = 1.36, SD = 0.886; M_soft_ = 1.44, SD = 0.852), and negative (M_sweet_ = 1.21, SD = 0.639; M_soft_ = 1.24, SD = 0.652) songs had the lowest corresponding ratings. Negative (M_bitter_ = 3.06, SD = 1.44) and hard (M_bitter_ = 2.73, SD = 1.43) songs evoked the most bitter ratings, followed by the soft (M_bitter_ = 1.32, SD = 0.75) and sweet (M_bitter_ = 1.15, SD = 0.51) songs. The negative song evoked the hardest (M_hard_ = 3.90, SD = 1.18) ratings, followed by the hard (M_hard_ = 2.82, SD = 1.40), the soft (M_hard_ = 1.61, SD = 0.87), and sweet (M_hard_ = 1.33, SD = 0.61) songs. Figure A1 summarizes the obtained cross-modal results.

A main effect of music was also found for the emotional ratings [F(6, 1072) = 83.33, *p* ≤ 0.001, partial eta squared = 0.318]. Multiple comparisons revealed that most differences in ratings were significant (*p* ≤ 0.005). The ratings that did not show any evidence of difference across participants were the negative ones between the positive/soft songs (*p* = 0.938). Concerning emotional ratings, the positive song was the one that evoked the most positive emotions (M_positive_ = 16.42, SD = 4.38), followed by the soft (M_positive_ = 13.09, SD = 4.67), the negative (M_positive_ = 10.19, SD = 4.64), and finally the hard (M_positive_ = 8.96, SD = 3.97) songs. The negative (M_negative_ = 14.88, SD = 5.12) and hard (M_negative_ = 14.84, SD = 4.79) songs evoked the most negative emotions, followed by the soft (M_negative_ = 6.64, SD = 3.08) and positive (M_negative_ = 5.50, SD = 1.47) songs. Figure A2 summarizes the emotional results obtained.
